# Lupeol and stigmasterol suppress tumor angiogenesis and inhibit cholangiocarcinoma growth in mice via downregulation of tumor necrosis factor-α

**DOI:** 10.1371/journal.pone.0189628

**Published:** 2017-12-12

**Authors:** Thaned Kangsamaksin, Supattra Chaithongyot, Chanida Wootthichairangsan, Rattanavinan Hanchaina, Chayada Tangshewinsirikul, Jisnuson Svasti

**Affiliations:** 1 Department of Biochemistry, Faculty of Science, Mahidol University, Bangkok, Thailand; 2 Division of Maternal Fetal Medicine, Department of Obstetrics and Gynecology, Faculty of Medicine Ramathibodi Hospital, Mahidol University, Bangkok, Thailand; 3 Laboratory of Biochemistry, Chulabhorn Research Institute, Bangkok, Thailand; University of South Alabama Mitchell Cancer Institute, UNITED STATES

## Abstract

Lupeol and stigmasterol, major phytosterols in various herbal plants, possess anti-inflammatory activities and have been proposed as candidates for anti-cancer agents, but their molecular mechanisms are still unclear. Here, we investigated the effects of lupeol and stigmasterol on tumor and endothelial cells in vitro and their anti-cancer activities in vivo. Our results demonstrated that lupeol and stigmasterol suppressed cell viability, migration, and morphogenesis of human umbilical vein endothelial cells (HUVECs) but not cholangiocarcinoma (CCA) cells. Expression analyses showed that the treatment of both compounds significantly reduced the transcript level of tumor necrosis factor-α (TNF-α), and Western blot analyses further revealed a decrease in downstream effector levels of VEGFR-2 signaling, including phosphorylated forms of Src, Akt, PCL, and FAK, which were rescued by TNF-α treatment. In vivo, lupeol and stigmasterol disrupted tumor angiogenesis and reduced the growth of CCA tumor xenografts. Immunohistochemical analyses confirmed a decrease in CD31-positive vessel content and macrophage recruitment upon treatment. These findings indicate that lupeol and stigmasterol effectively target tumor endothelial cells and suppress CCA tumor growth by their anti-inflammatory activities and are attractive candidates for anti-cancer treatment of CCA tumors.

## Introduction

Angiogenesis is a well-regulated process of new blood vessel formation from existing vasculature, that involves endothelial cell proliferation, migration, sprouting, lumen formation, and vessel maturation [1–3]. The formation of new vessels plays an essential role in development as well as pathological conditions, including cancer. Tumor angiogenesis, which is activated under hypoxic conditions, generates a network of new blood vessels that penetrate into growing tumors in order to supply oxygen and nutrients and remove waste products. Additionally, it is believed that tumor vasculature also provides a route for a small population of invasive tumor cells to travel to another site of the body during metastasis [4,5]. Therefore, most angiogenic signaling pathways and factors have become targets for the development of cancer therapeutics and proved to be somewhat successful [6,7]. Even though there is an incessant search for other angiogenic agents, many of which have been extensively investigated, most have failed in clinical trials or exhibited intolerably adverse side effects.

Phytosterols are plant sterols and stanols, whose structures are similar to cholesterol. They exist in several forms, including β-sitosterol, lupeol, campesterol, stigmasterol, stigmastanol, and cycloartenol. Legumes, cereal grains, and vegetable oils are found to be rich in naturally-occurring phytosterols. Several phytosterol-rich plants have been shown to possess a wide range of therapeutic activities [8]. Sterol-containing, ethanolic extracts from *Clinacanthus nutans* fresh leaves showed a significant inhibition on superoxide generation and elastase release by activated neutrophils, indicating anti-inflammatory and anti-oxidative actions [9]. Previous studies demonstrated that β-sitosterol increased the activities of anti-oxidant enzymes, superoxide dismutase, and glutathione peroxidase in macrophages under oxidative stress [10]. Treatment of β-sitosterol and campesterol significantly reduced production of prostaglandin E and prostaglandin I in liposaccharide-activated macrophage cells [11]. In addition, phytosterols have also been demonstrated to inhibit various types of cancer. For instance, consumption of phytosterols was found to be inversely related to stomach cancer incidence in humans [12]. β-sitosterol effectively reduced tumor growth in estrogen-responsive human breast cancer xenografts in mice [13]. The mechanism by which phytosterols inhibits tumor growth has been investigated in several studies. β-sitosterol promoted human leukemia cell apoptosis by increasing caspase-3 activities and inducing the Bax/Bcl-2 ratio [14]. A previous report demonstrated that β-sitosterol induced apoptosis in MCF-7 and MDA-MB-231 breast cancer cells via an increased level of Fas [15]. In addition, recent reports have demonstrated that campesterol significantly inhibited bFGF-induced angiogenesis in the chorioallantoic membrane of chicken eggs [16]. In contrast, β-sitosterol isolated from aloe vera was shown to possess angiogenic activities in vitro and has been recommended for management of chronic wounds [17]. Collectively, several lines of evidence showed that phytosterols in these herbal extracts represent the active compounds that possess beneficial activities for cancer therapeutic purposes; however, the effects on angiogenic activities are still unclear, and the mechanism of action remains elusive.

Here, we investigated the activities of three phytosterols: lupeol, β-sitosterol, and stigmasterol. They are the major phytosterols found in a wide range of Thai traditional medicinal herbs, including *C*. *nutans*. We analyzed in this study the action of the compounds on angiogenic processes in vitro and on tumorigenesis and tumor angiogenesis in vivo and found that lupeol and stigmasterol significantly inhibited HUVEC proliferation, migration, and network formation by downregulating TNF-α and inhibiting VEGF signaling in vitro, and the compounds effectively disrupted tumor angiogenesis, reduced macrophage recruitment, and suppressed the growth of cholangiocarcinoma.

## Materials and methods

### Cell lines and reagents

The study was approved by the Faculty of Medicine Ramathibodi Hospital Human Research Ethics Committee, Mahidol University (MURA2015/344) for the collection of umbilical cords. All cell cultures were maintained at 37°C in a mixture of 5% CO_2_ and 95% humidified air. Human umbilical vein endothelial cells (HUVECs) were isolated from umbilical cords as described [18] and grown in the Endothelial Cell Growth Media-2 media (EGM-2, Lonza) on a culture plate coated with rat tail type I collagen (BD Biosciences). The human cholangiocarcinoma cell line KKU-M213 was derived from the Japanese Collection of Research Bioresources Cell Bank, Japan (F1600039), and RMCCA-1 was a gift from the Tohtong laboratory [19]. Both cancer cell lines were maintained in 1X High Glucose DMEM (Invitrogen) with 10% fetal bovine serum (FBS) and 1X Pen-Strep (Invitrogen). Lupeol, β-sitosterol, and stigmasterol (Sigma-Aldrich) were prepared as stocks in DMSO and diluted to various concentrations in medium.

### Cell viability and migration assays

HUVECs were seeded in 96-well plates at a density of 3,000 cells/well and treated with compounds. The MTT assay was performed at 48 hours and measured at 540 nm for cell viability. To determine cell migration, HUVECs were seeded at 1.0 x 10^5^ cells per well in 24-well plates coated with type I collagen. After 24 hours when cells are confluent, the cell monolayer was scratched with a pipette tip across the diameter of each well. The medium and dislodged cells were removed, complete medium with compounds was then added to the plates, and cells were incubated at 37°C. Cell migration to the scratch area was photographed at 0- and 10-hour time points. The rate of cell migration was assessed using the TScratch program as described [20].

### Network formation assay

Porcine Collagen (Wako) was prepared on ice. 400 μl of collagen gel (Porcine Collagen: 10X RPMI 1640 medium: sterile water: Neutralizing Buffer; 7:1:1:1) was added to 24-well plates and incubated at 37°C for 1 hour. HUVECs were then overlaid at 1.0 x 10^5^ cells per well. After 2 hours when the cells completely adhered, the medium was removed, and another layer of collagen gel was added to the plates and incubated at 37°C for 3 hours. Then, complete medium with compounds was added to each well and changed every other day. Network formation and branching were photographed after 4 days.

### Quantitative real-time PCR (RT-PCR) and Western blot analysis

HUVECs were treated with different compounds in 60 mm dishes. For quantitative RT-PCR, RNA was collected using the GF-1 Total RNA Extraction Kit (Vivantis), and cDNA was synthesized with the RevertAid Reverse Transcriptase (Thermo Scientific). For Western blots, cells were lysed and probed following SDS/PAGE using antibodies from the Angiogenesis Sampler Kit (8696, Cell Signaling) and the anti-TNF-alpha antibody (3707; Cell Signaling). The TNF-alpha ELISA assay was performed using the TNF alpha Human ELISA Kit (KHC3011, Thermo Fisher Scientific). Briefly, HUVECs were treated with different compounds for 48 hours and changed to serum-free EBM-2 basal media (Lonza) for 12 hours before collection.

### In vivo toxicity analysis and tumor studies

The study was approved by the Faculty of Science, Mahidol University Animal Care and Use Committee (MUSC58-004-319). The experimental design for toxicity studies conformed to the guidelines and regulation in the US-FDA Redbook 2000: IV.C.3.a Short-Term Toxicity Studies with Rodents. C57BL/6 mice were administered with different doses of compounds via oral gavage 3 times a week for 28 days. Mice were monitored and blood and liver samples were collected at the end of the study for further analysis. For tumor studies, subcutaneous implantation of 1.0 x 10^6^ KKU-M213 cells and 7.5 x 10^5^ RMCCA-1 cells was performed in the upper left flank of nude mice. Two days later, compounds were administered through oral gavage 3 times a week. Animal conditions were monitored every other day for signs of sickness, including weight loss, appetite loss, and behavioral changes. Tumor size was smaller than 1 cm in diameter at the time of tumor collection. All animals were euthanized by CO_2_ inhalation and cervical dislocation, and tumors were harvested at day 28 and analyzed.

### Tissue analysis and immunohistochemistry

Fragments of tumor tissue were sharp dissected and homogenized with a homogenizer in RNeasy lysis buffer (Qiagen). RNA from the samples were prepared by using the RNeasy Mini Kit (Qiagen) and used for quantitative RT-PCR. The tumor tissue was also fixed in 10% formalin and paraffin-embedded for immunohistochemistry. Paraffin-embedded, fixed 5-μm sections were immunostained with hematoxylin and eosin, Masson’s trichrome, or primary antibodies: CD31 (MS-353-RQ, Thermo Scientific), F4/80 (ab6640, Abcam).

### Statistical analysis

All data are presented as means ± S.D. for all experiments and were analyzed using student’s t-tests for in vitro assays and one-way ANOVA for in vivo experiments.

## Results and discussion

### Effects of lupeol, β-sitosterol, and stigmasterol on endothelial cell viability

**Fig 1:**
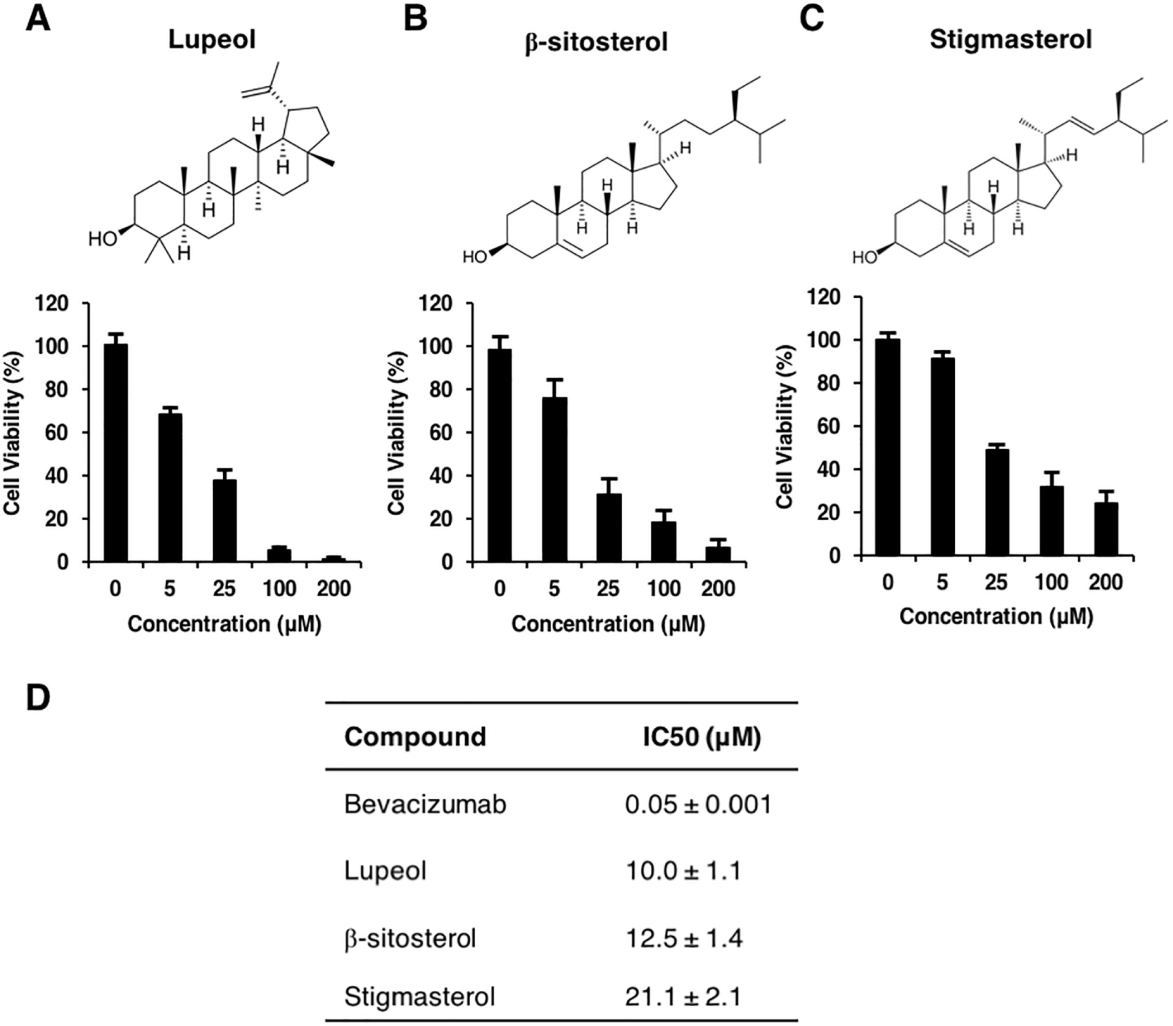
Lupeol, β-sitosterol, and stigmasterol exhibited cytotoxic effects on HUVECs. Structures and cell viability of lupeol (A), β-sitosterol (B), and stigmasterol (C). Cell viability of HUVECs, which were exposed for 48 hours to different doses of lupeol, β-sitosterol, and stigmasterol, as determined by MTT assays. Means ± S.D. *P value < 0.05 vs. control. n = 3. (D) The IC_50_ values of bevacizumab, lupeol, β-sitosterol, and stigmasterol in HUVECs. Means ± S.D.

First, we investigated the effects of lupeol, β-sitosterol, and stigmasterol (Fig 1A–1C) on the viability of human umbilical vein endothelial cells (HUVECs) isolated from the umbilical cord using the MTT assay and compare with bevacizumab as positive control. We found that these phytosterols showed similar dose-dependent toxic effects over a range of concentrations (Fig 1A–1C). The half inhibitory concentration (IC_50_) of lupeol, β-sitosterol, and stigmasterol were 10, 12.5, and 21.1 μM, respectively (Fig 1D). In contrast, we also determined their effects on cancer cells by using two intrahepatic cholangiocarcinoma cells, KKU-M213 and RMCCA-1, and an immortalized normal cholangiocyte, MMNK-1, and found that the IC_50_ on these cell lines were above 200 μM (S1 Fig). Therefore, these phytosterols exhibited a significantly higher toxic effect on endothelial cells than cancer and normal epithelial cells.

### Effects of lupeol, β-sitosterol, and stigmasterol on endothelial cell migration and capillary network formation

**Fig 2:**
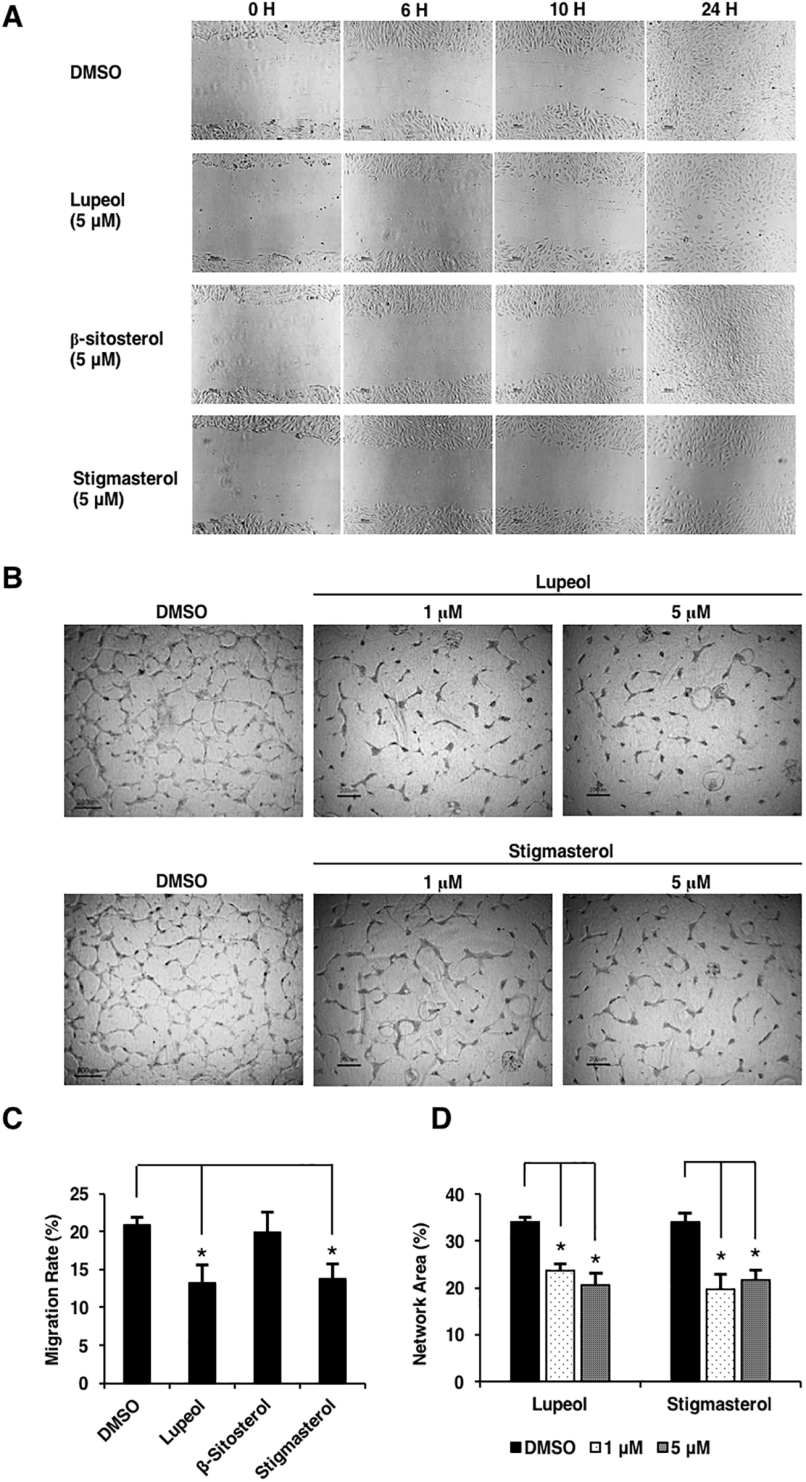
Lupeol and stigmasterol inhibited HUVEC migration and capillary network formation in vitro. (A) Lupeol and stigmasterol inhibited HUVEC migration. The effects of the compounds on HUVEC migration abilities were determined using 2-dimensional wound healing assays. HUVECs were seeded at 100% confluency and scratched at the middle of each well, then each compound was added to the culture at 5 μM concentration. Scale bar, 50 μm. (B) Lupeol and stigmasterol also significantly disrupted HUVEC network formation. Endothelial network formation assays were performed by seeding HUVECs between two type I collagen gel layers and cultured for 4 days. Scale bar, 200 μm.

In addition to cell proliferation, the processes of endothelial cell migration and network formation are also important for angiogenesis. We assessed the effects of the phytosterols on endothelial cell migration by wound healing assays and cell morphogenesis by determining the abilitiy of HUVECs to organize into capillary-like networks under the treatment of compounds at sub-lethal doses. In the wound healing experiment, HUVECs were seeded to 100 percent confluency and scratched in the middle of each well. The cells were allowed to migrate toward the wound in media containing each phytosterol at sub-lethal dose. The results showed that only lupeol and stigmasterol significantly reduced HUVEC migration when compared with negative control (Fig 2A). From these results, we further investigated whether these two phytosterols that inhibited both HUVEC viability and migration would also affect endothelial capillary network formation. HUVECs were seeded between two collagen gel layers and cultured in complete media for 48 hours. As shown in Fig 2B, both lupeol and stigmasterol significantly reduced endothelial network formation. The treatment of these compounds resulted in disconnected branches of cells and a reduction in the number of branch points. Both migration and network formation data were proved to be statistically significant (Fig 2C and 2D). As we performed these experiments under sub-lethal doses and, for wound healing assays, over the course of 24 hours, our results suggest that lupeol and stigmasterol inhibited endothelial cell migration and capillary network formation independent of their effects on cell proliferation or cell death.

### Lupeol and stigmasterol downregulated TNF-α and inhibited VEGF signaling

**Fig 3:**
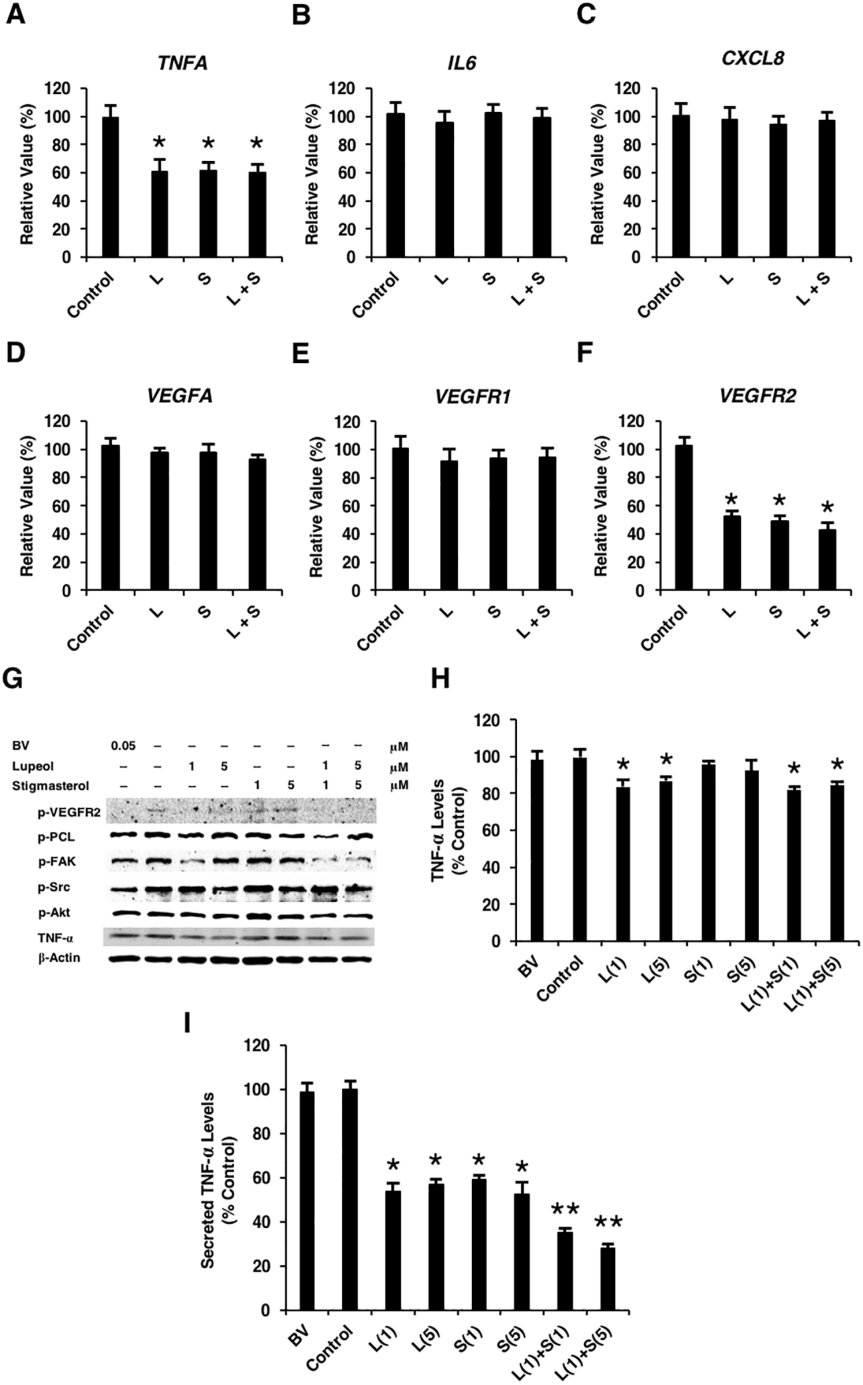
Lupeol and stigmasterol downregulated TNF-α and inhibited VEGF signaling. The expression of inflammatory cytokines and components of the VEGF pathway was examined by qRT-PCR in HUVECs treated with the compounds at 5 μM. (A-C) Lupeol and stigmasterol suppressed expression levels of TNF-α but showed no impact on IL-6 and CXCL-8. (D-F) Expression of VEGFR-2, but not VEGF-A or VEGFR-1, significantly decreased upon lupeol and stigmasterol treatment. (G) Western blotting showed a decrease in the p-FAK level at both concentrations and a slight decrease in levels of p-Src, p-PCL, p-Akt only at low concentration. L, lupeol; S; stigmasterol. (H) Densitometry calculations for TNF-αWestern blot data in (G) using ImageJ normalized to beta-actin. (I) ELISA for secreted TNF-alpha in the conditioned media from HUVECs treated with lupeol or stigmasterol or combination. Means ± S.D. *P value < 0.05 vs. control. n = 3. BV, bevacizumab; L, lupeol; S, stigmasterol; (1), 1 μM; (5), 5 μM.

Several lines of evidence suggest that phytosterols exhibit anti-inflammatory activities, so we investigated whether lupeol and stigmasterol had any impact on the expression of inflammatory cytokines in HUVECs. We found that the treatment of lupeol, stigmasterol, or both compounds significantly reduced the transcript levels of TNF-α (Fig 3A–3C) but did not affect expression of other inflammatory cytokines such as IL-6 and CXCL-8, suggesting that TNF-α may be a target of the anti-inflammatory activity of these phytosterols in endothelial cells. It has been demonstrated that TNF-α exerts a wide variety of biological effects, depending on cell type and microenvironment. In endothelial cells, TNF-α acts as an agonist for vascular endothelial cell activation by stimulating E-selectin, intercellular adhesion molecule-1 (ICAM-1), and IL-8 [21,22]. However, previous studies showed that TNF-α can promote or inhibit endothelial cell growth and angiogenesis depending upon cellular conditions [23]. Our results demonstrated that the treatment of lupeol or stigmasterol significantly decreased the transcript levels of VEGFR-2 but had no effect on those of VEGF-A or VEGFR-1 (Fig 3D–3F). Next, we further investigated the downstream effectors of the VEGF signaling pathway. As a positive control for VEGF signaling inhibition, bevacizumab significantly reduced levels of phosphorylated forms of VEGFR-2, Src, Akt, PCL, and FAK. Treatment of Lupeol, stigmasterol, or the combination significantly decreased the levels of p-VEGFR-2 and p-FAK (Fig 3G). We found that, similar to bevacizumab, both lupeol and stigmasterol exhibited mild inhibitory effects on Akt (Fig 3G). It is interesting to note that only low doses of lupeol and stigmasterol resulted in a decrease in p-PCL and p-FAK levels. As we observed a decrease in the TNF-α transcript, we investigated the protein level by Western blot analysis of HUVEC lysates and found that TNF-α was mildly reduced only under lupeol treatment (Fig 3G and 3H). We validated these findings by ELISA using conditioned media from HUVECs treated with the compounds. The level of secreted TNF-α was significantly decreased with the treatment of either lupeol or stigmasterol or the combination of both compounds (Fig 3I). These results suggest that both compounds disrupted endothelial cell viability, migration, and network formation by targeting TNF-α and VEGFR-2 signaling.

**Fig 4:**
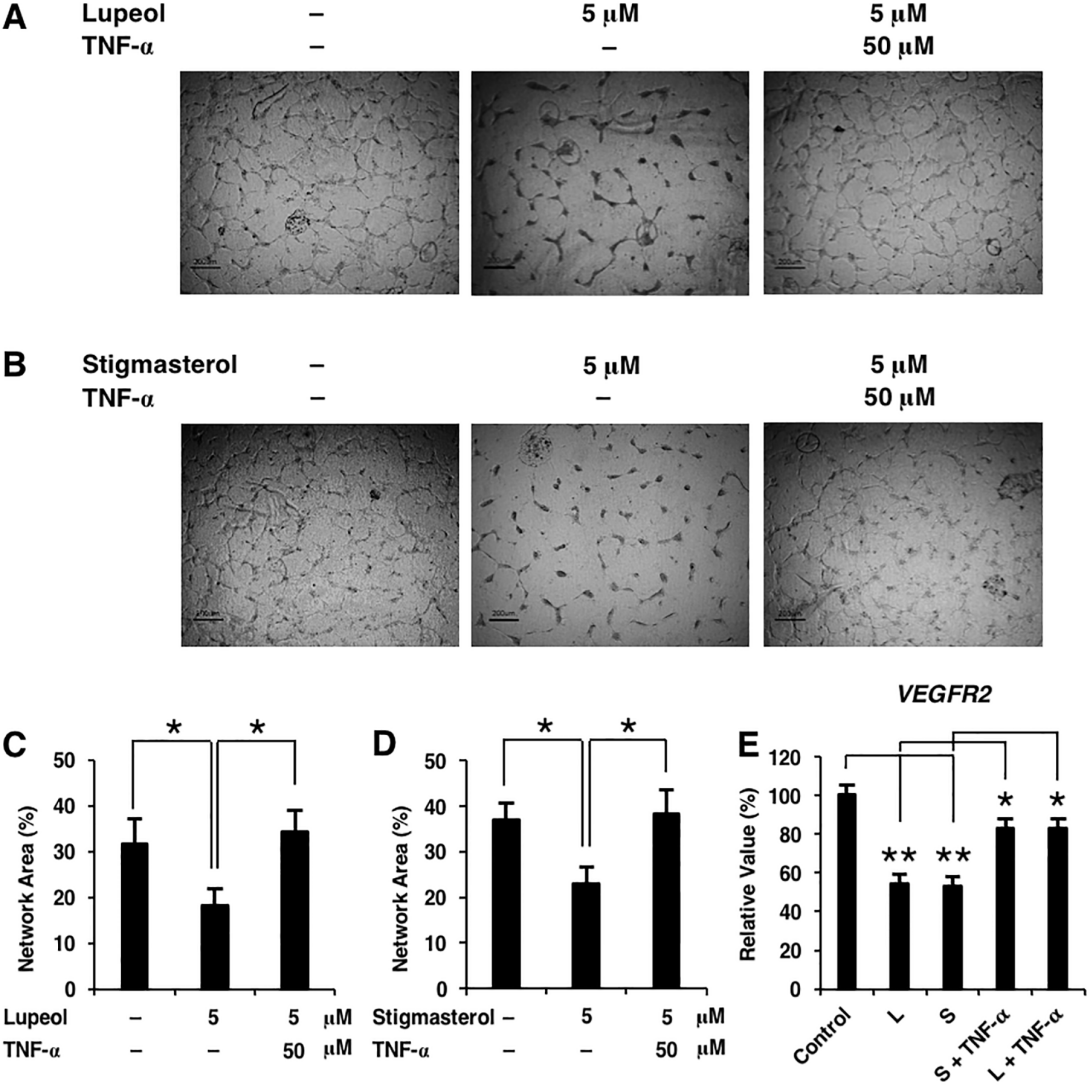
TNF-α suppression is necessary for the inhibitory effects of lupeol and stigmasterol on VEGFR-2 signaling and HUVEC network formation. (A-B) Endothelial network formation assays were performed upon the treatments of each compound at 5 μM with or without TNF-α. Addition of TNF-α drastically improved the ability of HUVEC to form endothelial network, similar to control. (C-D) Quantification of mean network area. (E) Lupeol or stigmasterol treatment suppressed VEGFR-2 expression, which could be rescued by the addition of recombinant TNF-α. Data presented ± S.D. *P value < 0.05, ** P value < 0.01 (n = 4–5).

We further investigated whether downregulation of TNF-α was necessary for the suppression of VEGFR-2 signals. As previously shown, lupeol and stigmasterol significantly disrupted HUVEC capillary network formation (Fig 2B and 2D). Our results showed that such effects were abolished when TNF-α was added to the culture (Fig 4A–4D), suggesting that a decrease in TNF-α levels was necessary for the anti-angiogenic activity of the phytosterols. Then, we determined that the transcript levels of VEGFR-2 were rescued upon treatment of the compounds and TNF-α (Fig 4E). These results confirmed that lupeol and stigmasterol disrupted in vitro angiogenesis by downregulating TNF-α, which subsequently inhibited VEGFR-2 expression and VEGF signaling and suppressed endothelial cell morphogenesis in vitro.

### Lupeol and stigmasterol did not exhibit significant toxic effects in mice

**Fig 5:**
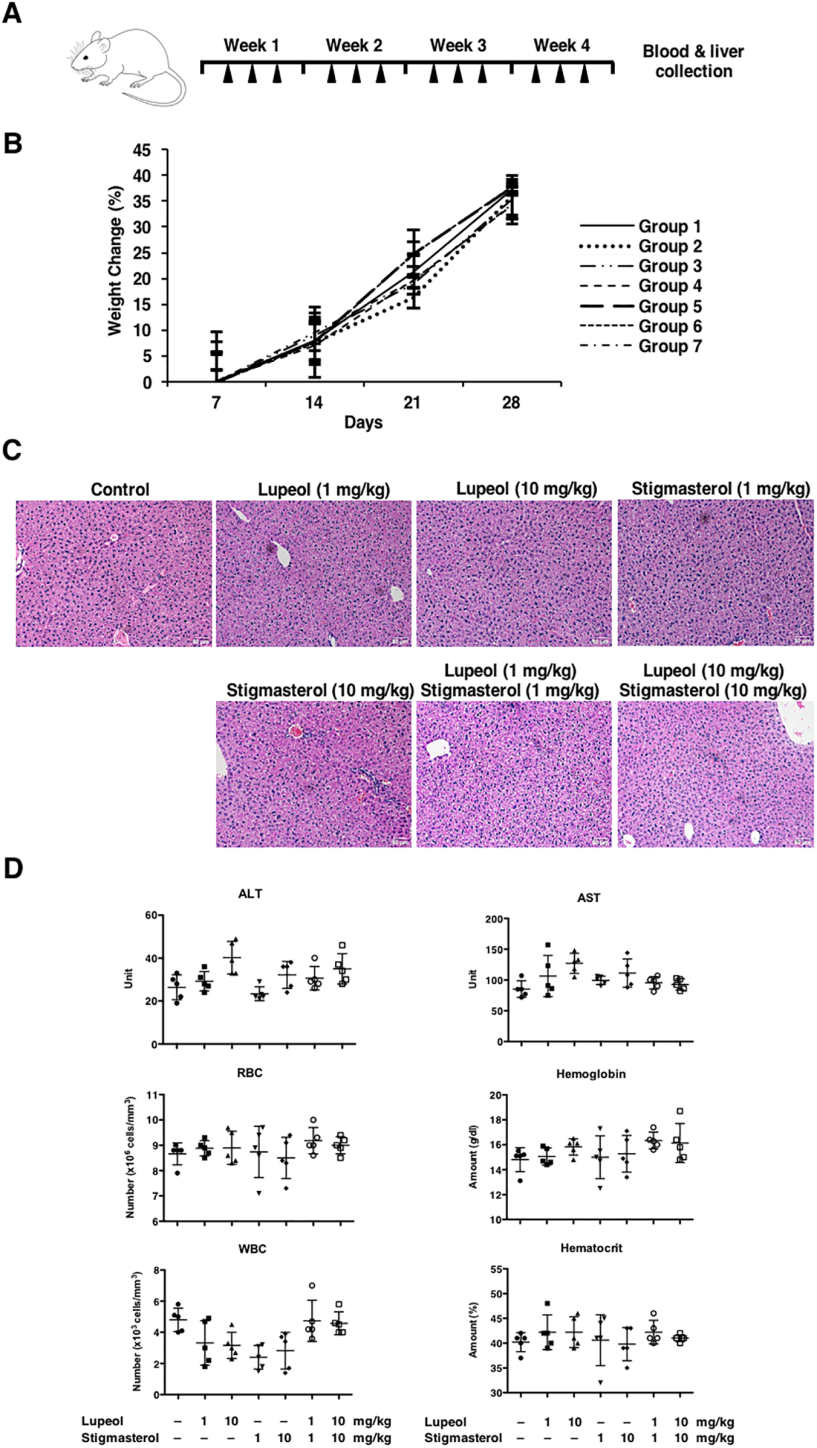
Lupeol and stigmasterol did not exhibit significant toxic effects in mice. (A) Treatment timeline for the toxicity studies. (B) No significant difference in weight change was observed between the control and treatment groups. Data presented as percent weight mean change ± S.D. (n = 5). (C) H&E staining of the livers showed no difference between the control and treatment groups. (D) Sera were also collected from mice for blood chemistry analyses. No significant differences were observed in ALT, AST, RBC, Hemoglobin, WBC, Hematocrit using one-way ANOVA. Group 1, control; Group 2, lupeol (1 mg/kg); Group 3, lupeol (10 mg/kg); Group 4, stigmasterol (1 mg/kg); Group 5, stigmasterol (10 mg/kg); Group 6, lupeol + stigmasterol (1 mg/kg); Group 7, lupeol + stigmasterol (10 mg/kg).

Next, we investigated the toxicity of these compounds in mice by treating each compound or a combination of both three times a week for four weeks via oral gavage at 1 mg/kg or 10 mg/kg doses (Fig 5A). We found that there were no significant weight or behavioral changes observed (Fig 5B). In addition, histopathologic analysis of the livers of mice treated with the compounds demonstrated no signs of liver toxicities, and there were no significant effects on levels of markers of systemic and hepatic damage from serum analyses (Fig 5C and 5D), suggesting that both lupeol and stigmasterol can be safely used in animals.

### Treatment of lupeol and stigmasterol reduced tumor angiogenesis, tumor growth, and macrophage recruitment in cholangiocarcinoma xenograft models

**Fig 6:**
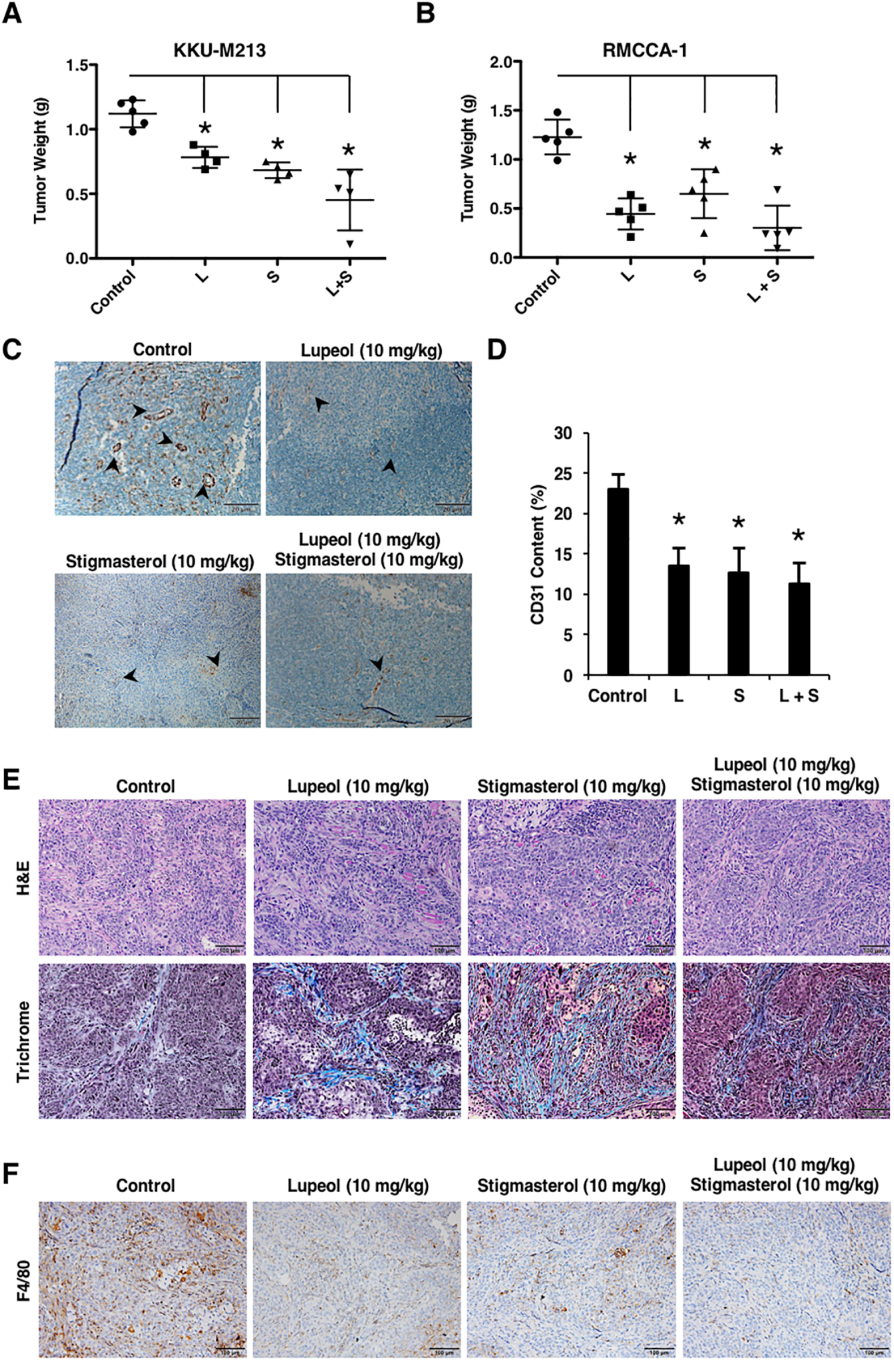
Treatment of lupeol and stigmasterol reduced tumor angiogenesis, tumor growth, and macrophage recruitment in cholangiocarcinoma xenograft models. (A) KKU-M213 and (B) RMCCA-1 tumors weighed less in the lupeol or stigmasterol, or combination treatment groups compared with control. Data, as mean tumor weight ± SD; *P value < 0.05 (n = 4–5). (C) CD31 staining of compound-treated KKU-M213 tumors; scale bar, 200 μm. (D) Quantification of CD31-positive areas. Data, mean percentage of the CD31-positive area ± SD; *P < 0.003 (n = 4–5). (E) H&E, Masson’s trichrome, and (F) F4/80 staining of KKU-M213 tumors. Scale bar, 200 μm. L, lupeol; S; stigmasterol.

The anti-tumor and anti-angiogenic activities of lupeol and stigmasterol were investigated using human cholangiocarcinoma xenograft models in nude mice (S2 Fig). As the compounds exhibited little or no toxic effects on CCA cells in vitro but effectively reduced the viability and morphogenesis of endothelial cells, we hypothesized that both lupeol and stigmasterol would suppress tumor growth by disrupting the growth and migration of the host endothelial cells and therefore preventing functional tumor angiogenesis. We treated mice bearing CCA tumor xenografts of approximately 100 mm^3^ with 10 mg/kg lupeol or stigmasterol or a combination of both compounds 3 times a week for 4 weeks. Our results demonstrated that both single and dual treatment significantly delayed CCA tumor growth by 28% (lupeol), 50% (stigmasterol), and 59% (combination) (Fig 6A and 6B). Next, we investigated the effects of the compounds on tumor angiogenesis by CD31 immunohistochemistry and found that both lupeol and stigmasterol significantly reduced CD31-positive endothelial content by 41%, 46%, and 51%, respectively, and disrupted tumor vasculature (Fig 6C and 6D), indicating that both compounds have similar mechanisms of action. It has been previously demonstrated that CCAs are usually characterized by a prominent desmoplastic stroma, and the fibrotic components may have an important impact in tumor angiogenesis and tumor growth [24], and previous reports showed that stromal desmoplasia acts to restrain the growth of certain types of cancer, including pancreatic ductal carcinoma [25]. Our Masson’s trichrome staining of CCA tumors was consistent with previous reports and revealed overt collagen fibrotic deposition (Fig 6E and S3 Fig), which generally correlates with increased tissue necrosis due to the inhibition of tumor cell growth and proliferation. In addtion, F4/80 immunohistochemistry showed that a decreased population of macrophages were recruited to the tumor area upon treatment (Fig 6F and S4 Fig). These results were consistent with our in vitro data and indicated that lupeol and stigmasterol may cause the decreased production of chemokines, and consequently led to a decrease in macrophage recruitment. We next investigated whether the treatment of the compounds affected inflammatory cytokine production and found that treatment of lupeol or stigmasterol decreased IL-6, CXCL-8, and TNF-α levels in tumor tissues (S4 Fig). This is not surprising to us because in our in vivo studies the sources of inflammatory cytokines were not limited to endothelial cells, and cellular components in the stroma also contribute to cytokine levels in tissue. Therefore, our results indicated that lupeol and stigmasterol may also act on stromal cells in downregulating the expression of inflammatory cytokines and, consequently, disrupt tumor angiogenesis and suppress tumor growth.

## Conclusions

Links between inflammation and cancer were first established on the basis of the fact that the presence of inflammation and inflammatory cells was correlated with tumor characteristics. Epidemiological evidence has also shown that chronic inflammation predisposes humans to various types of cancer [26]. Therefore, there has been a continuous search for anti-inflammatory agents and an extensive study of the use of such agents as anti-cancer therapeutics. Several lines of evidence have demonstrated that key molecules that are involved in cancer-related inflammation include NF-kB, STAT3, and major inflammatory cytokines, such as IL-1b, IL-6, IL-23, and TNF-a [27–32]. Leukocyte infiltration is also correlated with angiogenesis, which is necessary for the growth of solid tumors. Tumor-associated macrophages (TAMs) with the M2 phenotype have been shown to be involved in promoting tumor progression, angiogenesis, and suppressing adaptive immunity. However, the molecular mechanisms of pro- and anti-angiogenic inflammatory cytokines are still not completely understood.

Here, our findings provide evidence that both lupeol and stigmasterol exert anti-angiogenic activities that can effectively suppress tumor growth in vivo. As they exhibited little impact on the viability of normal epithelial cells or tumor cells, these compounds may be beneficial in cancer therapy by selectively targeting tumor endothelial cells without possible side effects on normal cells. Moreover, this study discovered the role of TNF-α in promoting endothelial cell morphogenesis and angiogenesis by its regulation of VEGFR-2 expression in HUVECs. Our data demonstrated a link between inflammatory responses and angiogenic processes. While inhibition of VEGF signaling has already been established, and several anti-angiogenic agents have been clinically approved by the US FDA, such as bevacizumab and sorafenib, our data showed that lupeol and stigmasterol acts to inhibit the VEGF pathway by downregulating TNF-α and may have some unknown non-overlapping mechanisms of action that preferentially target certain downstream effectors, especially focal adhesion kinase, indicating that these compounds may provide additional anti-angiogenic activities through their unique activity.

Additionally, it is interesting to see that while the activity of the compounds was specific to endothelial cells, our tissue analysis indicated that lupeol and stigmasterol, as anti-inflammatory agents, also had an impact on stromal cells that may have enhanced the anti-angiogenic effect that we observed in the tumor studies. These data provide evidence that inflammatory cytokines from both tumor cells and stromal cells play an important role in tumor angiogenesis and tumor growth in cholangiocarcinoma. And, targeting tumor cells, tumor endothelium, and tumor stroma may promote the efficacy of anti-cancer therapeutics in CCA and other stroma-rich cancers as well.

In conclusion, our study demonstrates that lupeol and stigmasterol are anti-angiogenic compounds that inhibit endothelial cell proliferation, migration, and capillary network formation through the disruption of the TNF-α-VEGFR-2 axis, and they effectively suppress the growth cholangiocarcinoma xenografts by downregulating inflammatory cytokine production, macrophage recruitment and tumor angiogenesis. This study provides for the first time the evidence that demonstrates the importance of the TNF-α-VEGFR-2 axis in endothelial morphogenesis and tumor growth.

## Supporting information

S1 Fig Lupeol, β-sitosterol, and stigmasterol showed minimal cytotoxic effects on cholangiocarcinoma cells, KKU-M213 and RMCCA-1, and normal cholangiocytes, MMNK-1. (A-C) Cell viability of cholangiocarcinoma cells or cholangiocytes was measured by the MTT assays upon the compound treatments at concentrations ranging from 5 to 200 μM. Data presented ± S.D. (n = 5).
(TIFF)

S2 Fig Treatment of lupeol and stigmasterol reduced tumor growth in cholangiocarcinoma xenograft models. Tumor size in (A) KKU-M213 and (B) RMCCA-1 xenograft models was significantly reduced upon treatment of the compounds.
(TIFF)

S3 Fig Treatment of lupeol and stigmasterol significantly increased stromal content but decreased macrophage recruitment in cholangiocarcinoma tumors. Quantification of (A) Masson’s trichrome- and (B) F4/80-positive areas was performed. Data, mean percentage of the positive area ± SD; *P < 0.005 (n = 4–5). L, lupeol; S; stigmasterol.
(TIFF)

S4 Fig Treatment of lupeol and stigmasterol reduced expression of inflammatory cytokines IL6, IL12, and TNF-α. The expression levels of (A) IL6, (B) IL12, (C) CXCL8, (D) TNFA were determined by quantitative RT-PCR in tumor tissue samples. Data presented ± S.D. (n = 4–5). L, lupeol; S; stigmasterol.
(TIFF)
