# Supplementary figures and images for: Lupeol and stigmasterol suppress tumor angiogenesis and inhibit cholangiocarcinoma growth in mice via downregulation of tumor necrosis factor-α
Source: PLoS One. 2017 Dec 12;12(12):e0189628. doi: 10.1371/journal.pone.0189628 (PMC5726636; doi:10.1371/journal.pone.0189628)

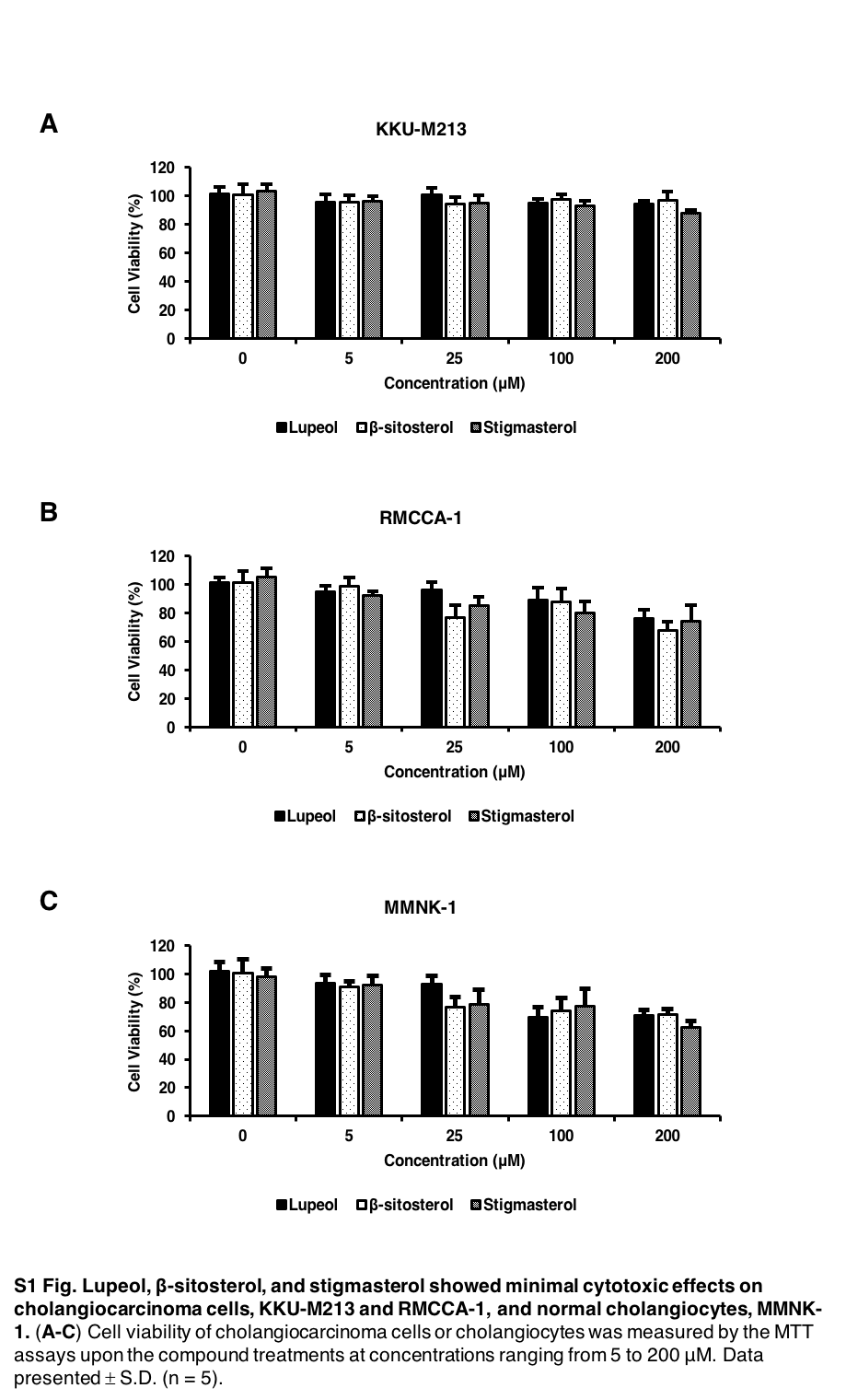

Supplement: S1 Fig — (A-C) Cell viability of cholangiocarcinoma cells or cholangiocytes was measured by the MTT assays upon the compound treatments at concentrations ranging from 5 to 200 μM. Data presented ± S.D. (n = 5). (TIFF) [file pone.0189628.s001.tiff]

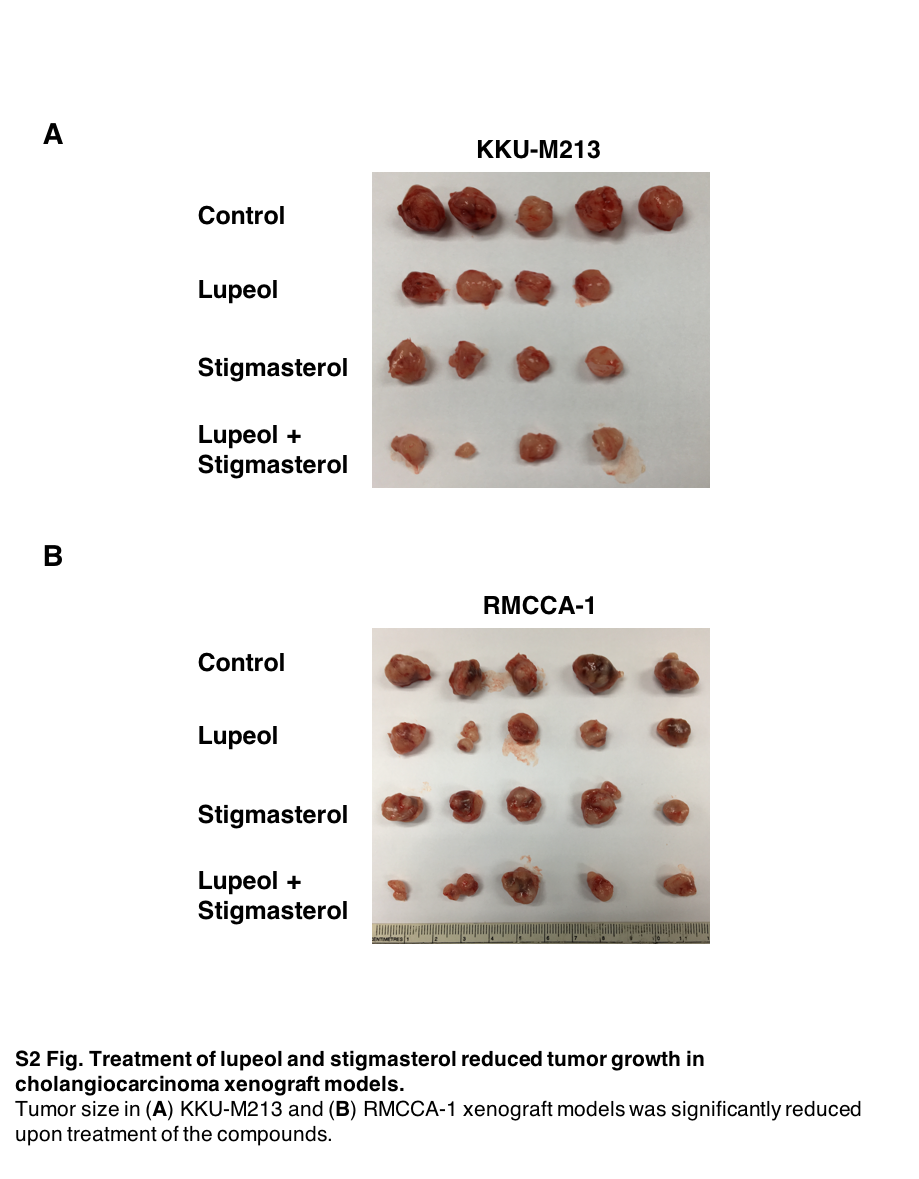

Supplement: S2 Fig — Tumor size in (A) KKU-M213 and (B) RMCCA-1 xenograft models was significantly reduced upon treatment of the compounds. (TIFF) [file pone.0189628.s002.tiff]

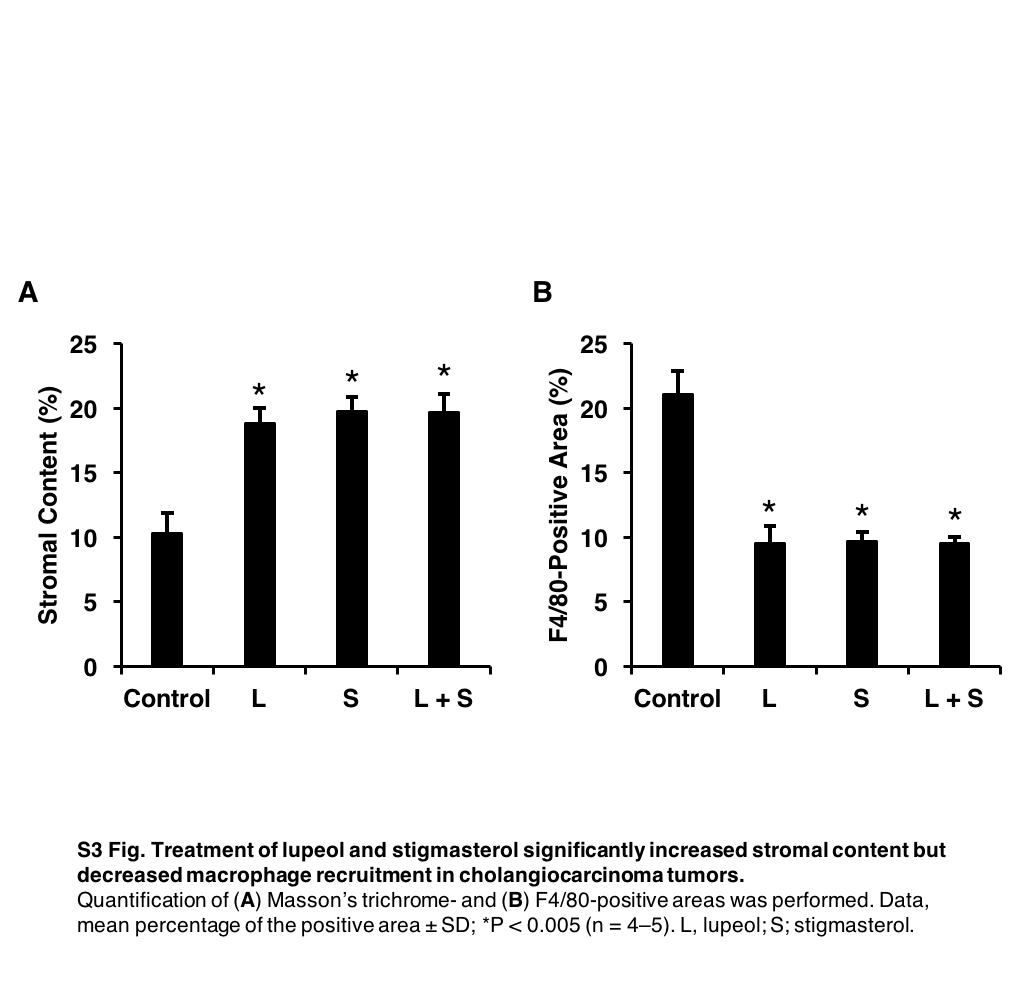

Supplement: S3 Fig — Quantification of (A) Masson’s trichrome- and (B) F4/80-positive areas was performed. Data, mean percentage of the positive area ± SD; *P < 0.005 (n = 4–5). L, lupeol; S; stigmasterol. (TIFF) [file pone.0189628.s003.tiff]

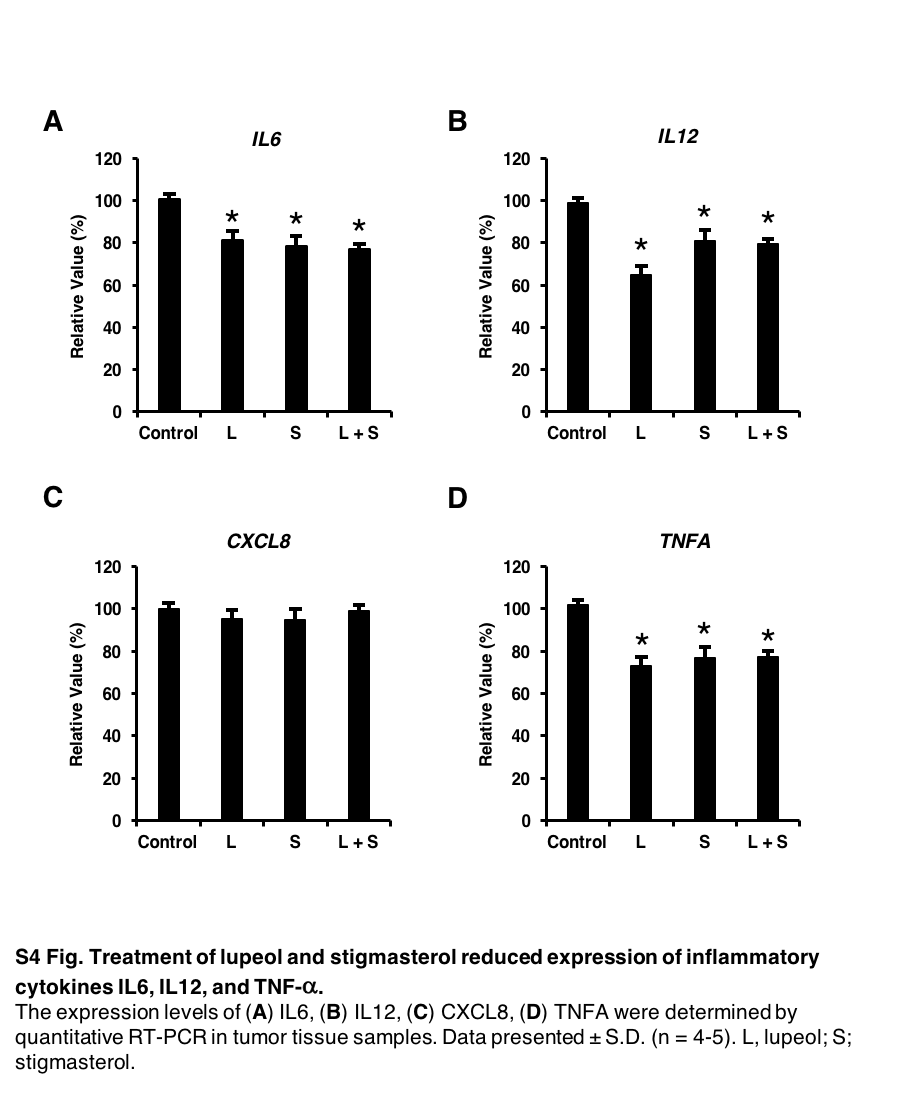

Supplement: S4 Fig — The expression levels of (A) IL6, (B) IL12, (C) CXCL8, (D) TNFA were determined by quantitative RT-PCR in tumor tissue samples. Data presented ± S.D. (n = 4–5). L, lupeol; S; stigmasterol. (TIFF) [file pone.0189628.s004.tiff]
